# The Role of Renal Macrophage, AIM, and TGF-β1 Expression in Renal Fibrosis Progression in IgAN Patients

**DOI:** 10.3389/fimmu.2021.646650

**Published:** 2021-06-14

**Authors:** Min Yang, Jia Wei Liu, Yu Ting Zhang, Gang Wu

**Affiliations:** ^1^ Renal Division of Northern Jiangsu People’s Hospital, Clinical Medicine College of Yangzhou University, Yangzhou, China; ^2^ Renal Division of Xi’an People’s Hospital, Xi’an, China; ^3^ Intensive Care Unit of The Second Clinical Medical College of Nanjing Medical University, Nanjing, China

**Keywords:** IgAN, renal fibrosis, macrophage, AIM, TGF-β1

## Abstract

**Objective:**

To analyze the expression of macrophages, AIM, TGF-β1 in the kidney of IgAN patients, and to explore the role of macrophages, AIM, TGF-β1 in the progression of renal fibrosis in IgAN patients.

**Methods:**

The paraffin specimens of renal tissue from 40 IgAN patients were selected as the observation group. At the same time, paraffin specimens of normal renal tissue from 11 patients treated by nephrectomy were selected as the normal control group. We observed the distribution of macrophages, the expression of AIM and TGF-β1 by immunohistochemical staining and/or immunofluorescence.

**Result:**

The number of M0, M1, M2 macrophages could be found increased in IgAN patients. M0 macrophages are mainly polarized towards M2 macrophages. The expression of AIM and TGF-β1 were significantly higher in IgAN patients than in NC. M2 macrophage, AIM and TGF-β1 were positively correlated with serum creatinine and 24-hour proteinuria, but negatively correlated with eGFR. M2 macrophages, AIM, TGF-β1 were positively correlated with fibrotic area.

**Conclusion:**

M2 macrophages, AIM and TGF-β1 play important roles in the process of IgAN fibrosis, and the three influence each other.

## Introduction

IgA nephropathy (IgAN) is a common systemic immune glomerulonephritis, which is characterized by the deposition of IgA or IgA-based immune complexes in the mesangial region with mesangial cell proliferation and mesangial matrix expansion (1, 2). Although IgAN progresses slowly, up to 50% of patients develop to end-stage, and it is the main type that causes ESRD (3). Renal fibrosis is the common pathway in the progression of chronic kidney disease (CKD) (4). In recent years, it is found that macrophages, Apoptosis inhibitor of macrophage (AIM) and transforming growth factor-β1 (TGF-β1) all play important roles in renal fibrosis.

Macrophages are involved in the development of many kidney diseases. Some scholars believe that the deposition of macrophages in the kidney can be used as an important indicator to judge the development and prognosis of renal diseases. Macrophages are divided into M0, M1 and M2 types (5). M1 macrophages promote Thl type inflammatory response and remove bacteria or tumor cells by secreting inflammatory factors such as IL-1, IL-6, IL-12, TNF-α, Reactive oxygen species(ROS) and NO. However, if the inflammation persists, it will further spread and eventually lead to tissue fibrosis (6). By expressing Arginase 1 (Arg1), Chitinase-like 3 protein (Ym1) and hypoxia-induced mitogenic factor (HIMF) to resist the stimulation of pathogenic microorganisms and allergens, M2 macrophages limit inflammatory response and type I adaptive immunity, eliminate residue, promote angiogenesis, play an anti-inflammatory role, reduce cell apoptosis, promote cell proliferation, and promote tissue repair (7, 8). Exactly, what type of macrophage plays a major role in renal fibrosis of IgAN is still controversial. Studies showed that CD68 and CD80 can be used as specific surface markers of M0 and M1 macrophages (9); CD163 is a highly specific mannose receptor expressed in M2 macrophages, which is not expressed in M1 macrophages. Thus CD68, CD80 and CD163 can be used as markers to represent the presence of different types of macrophages (10–12).

In addition, AIM was originally discovered as a secreted protein of macrophages and thus was named Spa (13). It was later named AIM after its antiapoptotic effect on white blood cells was found (14), or CD5L according to the human genome organization nomenclature. AIM has a wide range of functions and plays an important role in regulating leukocyte migration balance, metabolism and inflammatory response. Many literatures have mentioned that AIM plays a role in immune inflammatory response (15), lipid homeostasis, non-alcoholic liver disease (16), autoimmune disease, atherosclerosis (17) and other diseases. In recent years, there have been numerous studies on the role of AIM in renal disease. For example, Tadashi Uramat et al. (18) found in the hypertensive prone mouse model that reducing the expression of AIM and oxLDL(oxidized low-density lipoprotein, which have the effect of up-regulating the expression of AIM) can effectively reduce the fibrosis of renal tissue. Similarly, Megumi Oshima et al. (19) found that the area of AIM and macrophage deposition in renal tissue was positively correlated with the severity of proteinuria and eGFR decrease in patients with CKD. All the above studies suggest that AIM also plays an important role in the process of renal fibrosis. However, opposite results exist at present: It has been found in the acute kidney injury model that AIM can promote the removal of apoptotic cell debris by renal tubular epithelial cells (20).

At present, many studies have elaborated on the role of macrophages, AIM, TGF-β1 in renal disease, but there are few studies on the role of the three in the progress of IgAN, fibrosis process and the correlation among the three. If the pathogenesis of the progression of IgAN fibrosis can be clarified, it may open a new window for the treatment and prognosis of IgAN. To investigate the role of macrophages, AIM, TGF-β1 in the progression of IgAN fibrosis, we detected infiltration of macrophages in renal tissues of IgAN patients and the expression level and characteristics of AIM and TGF-β1, and then analyzed the relationship among the three and clinical related indicators and renal fibrosis area of IgAN patients.

## Methods

### Subjects

Forty patients with IgAN confirmed *via* renal biopsy were recruited from Subei People Hospital. Patients with chronic systemic diseases (systemic lupus erythematosus, diabetes mellitus, Henoch-Schönlein purpura, liver cirrhosis, etc.), (treatment of glucocorticoid, immunosuppressor, ACEI, ARB, etc.), or advanced renal failure (estimated glomerular filtration rate (eGFR) ≤15ml/min/1.73m2) were excluded. eGFR levels were calculated using the Modification of Diet in Renal Disease (MDRD) equation. Clinical information (gender, age, weight, disease history) and laboratory data (serum albumin, serum creatinine, urine red blood cell, 24 h urinary protein and eGFR levels) were collected at the time of biopsy. Histologically normal kidney tissues dissected adjacent to renal tumor were used as controls (n=11). Written informed consent was obtained from all subjects and the study was approved by the Ethics Committee of Subei People Hospital.

### Immunofluorescent and Immunohistochemical Staining

Human kidney tissue sections were analyzed with immunohistochemical and immunofluorescent staining. Masson staining was performed on all human kidney tissue sections. The sections were deparaffinized and treated with 3% hydrogen dioxide. After antigen retrieval and blocking, sections were incubated overnight with a human CD68 antibody (diluted 1:600, Abcam), human CD80 antibody (diluted 1:600, Abcam), human CD163 antibody (diluted 1:600, Abcam), human AIM antibody (diluted 1:150, Abcam) or a human TGF-β1 antibody (diluted 1:500, Abcam). After treatment with a horseradish peroxidase-labeled biotin-conjugated secondary antibody (diluted 1:200, Biosynthesis Biotechnology, China) and DAB staining (ZSGB-Bio, China), sections were observed under a microscope. 10 randomly selected fields (400×) were imaged per section. The expression of macrophages in glomeruli and tubules was quantified using Image-Pro Plus Image Analysis Software (Meyer Instruments, Inc., Houston, TX, USA); expressions of AIM and TGF-β1 in glomeruli were quantified in the same manner. The integrated optical density (IOD) was measured for each image by two pathological experts independently. The average IOD/positively stained area (AIOD) was then calculated. Macrophages, AIM and TGF-β1 expression levels in IgAN renal biopsies and normal kidney biopsies were compared by calculating the AIOD. Nuclei were stained with DAPI at room temperature for 5 min. Images of fluorescently labeled sections were obtained using a fluorescent microscope (ZEISS, Axioimager.Z2).

### Masson Staining for Fibrosis Assessment

The fibrosis area was green, and we calculated the percentage of the green area in the visual field area to reflect the degree of fibrosis. Three high-magnification fields were randomly selected for each section (All specimens were photographed under the same conditions), avoiding the large vessels. IPP6.0 image analysis system was used to statistically analyze the area ratio of the fibrosis area (All specimens were analyzed in the same manner).

### Statistical Analyses

Statistical analysis was performed using SPSS 22.0. The data were expressed as mean ± SD or as medians (range). Significant differences were assessed using either at-test or one-way ANOVA. A nonparametric Mann-Whitney U test was performed to compare the integrated optical density between experimental groups. A two-tailed P<0.05 was considered statistically significant.

## Results

### Distribution of Macrophages in the Kidney

**Figure 1:**
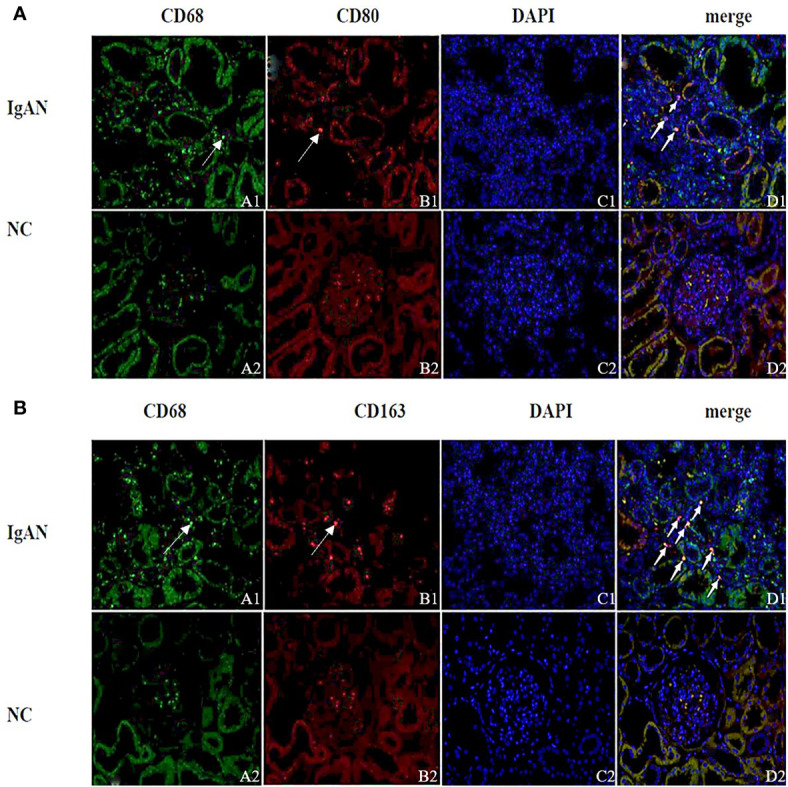
Distribution of different macrophages in the renal interstitium. **(A)** Immunofluorescence double staining of CD68 and CD80 (A1 showed CD68^+^ cells (green), B1 showed CD80+ cells (red), D1 showed CD68^+^CD80^+^ cells (yellow), and M0 polarized towards M1). **(B)** Immunofluorescence double staining of CD68 and CD163 (A1 shows CD68^+^ cells (green), B1 shows CD163^+^ cells (red), D1 shows CD68^+^CD163^+^ cells (yellow), M0 polarization towards M2). (Original magnification×400) PS: **(A, B)** A small amount of red blood cells was nonspecific stained in the glomeruli of the NC group (None nucleus after merge, DAPI^-^); Cell nuclei were stained blue with DAPI.

**Figure 2:**
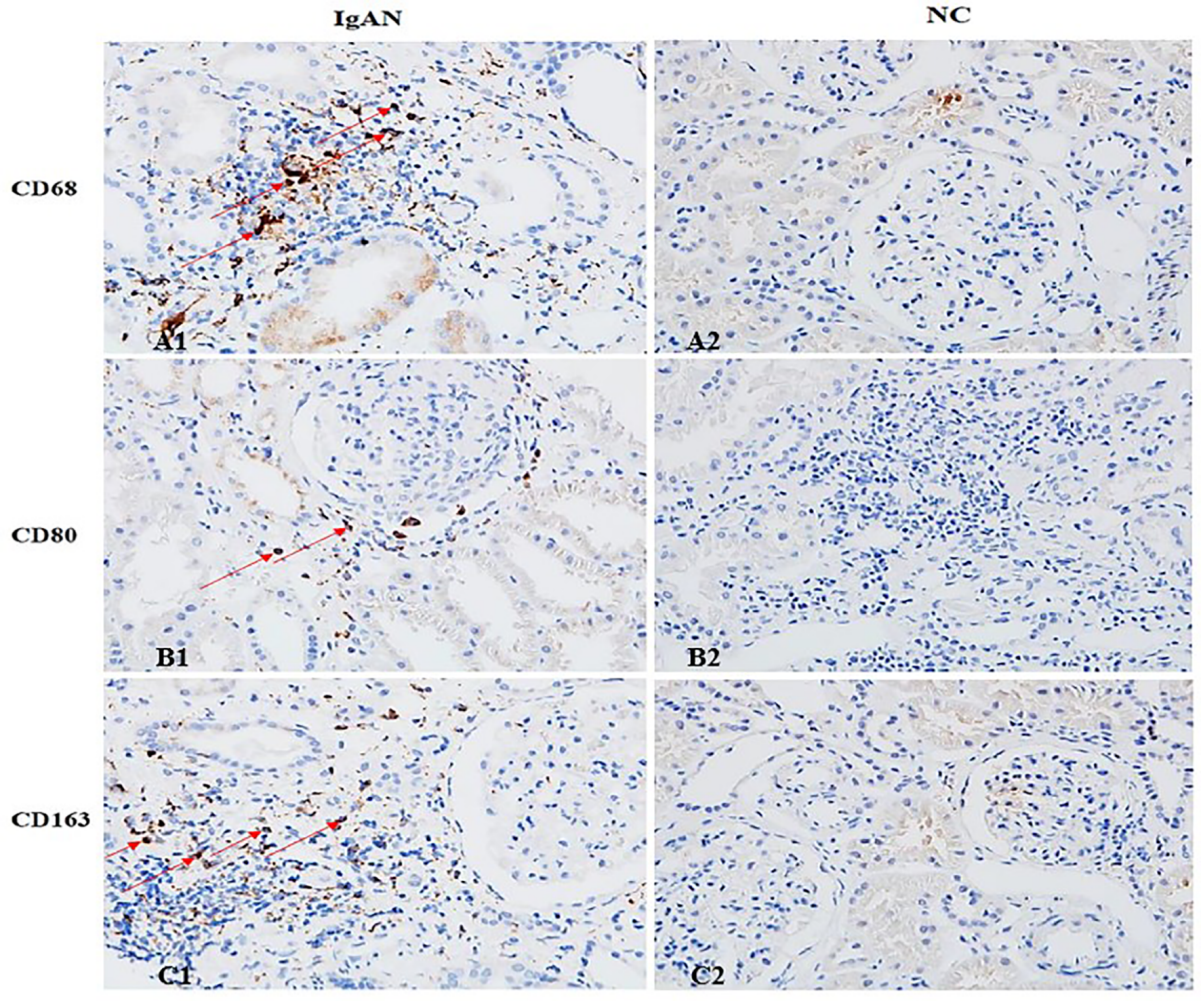
The distribution of different macrophages in the renal interstitium. M0 (CD68), M1 (CD80) and M2 (CD163) macrophages in IgAN group were mainly distributed in the renal interstitium (Cytoplasm brown was the positive macrophage). There were occasional or no macrophages in the interstitium of renal tissue in the NC group. (A1 showed CD68^+^ cells, B1 showed CD80^+^ cells, and C1 showed CD163^+^ cells). (Original magnification×400).

**Figure 3:**
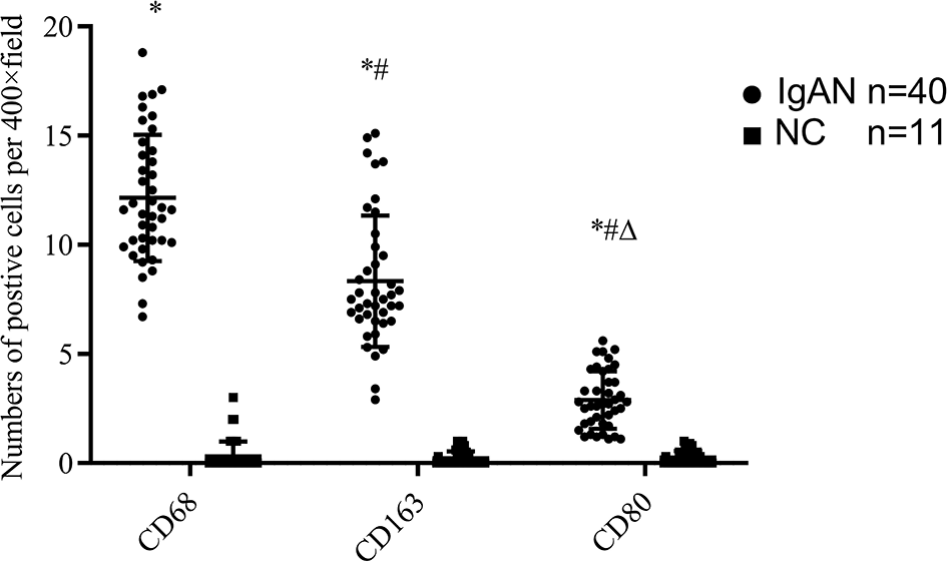
The expressions of different macrophages. The positive expressions of M0 (CD68), M1 (CD80) and M2 (CD163) in renal macrophages showed statistically significant differences between IgAN and NC. *P<0.0001, IgAN *vs*. NC; #P < 0.0001, CD163/CD80 *vs*. CD68; △P < 0.0001, CD163 *vs*. CD68. The positive expression of CD163 was higher than that of CD80, and the polarization of M0 towards M2 was dominant.

Immunofluorescence and immunohistochemistry (**Figures 1**, **2**) showed that macrophages in the IgAN group were mainly distributed in the renal tubular interstitial, and occasionally in the glomerular capillary plexus and renal tubular lumen, while macrophages were not distributed in normal renal tissue. Moreover, the number of M0, M1 and M2 macrophages in the renal interstitium of IgAN was different, and there was more polarization of M0 towards M2 (P*<*0.05, **Figure 3**).

### Expression of AIM, TGF-β1 in Renal Tissue

**Figure 4:**
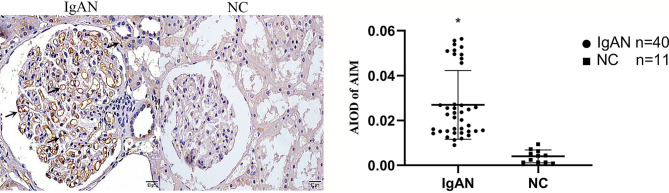
Expression of AIM in two groups. AIM mainly expressed in glomerular capillary loops and renal tubular epithelial cells in renal tubular lumen in IgAN group, but infrequent or absent in NC group. (Original magnification×400); *P<0.05, IgAN *vs*. NC.

**Figure 5:**
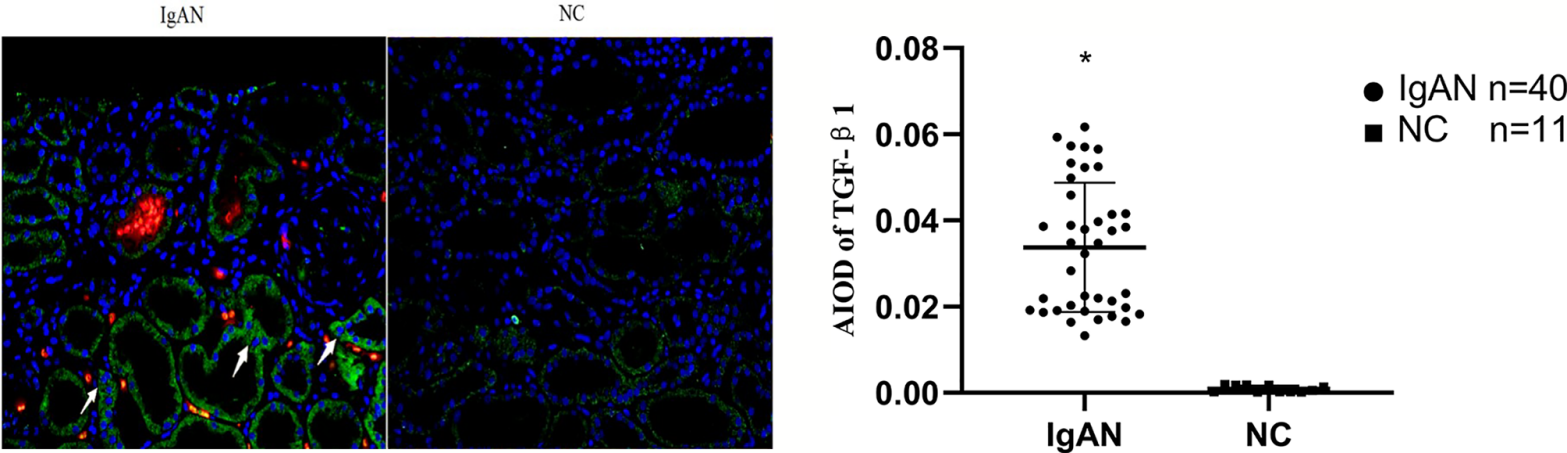
Expression of TGF-β1 in two groups. TGF-β1 expressed mainly in renal tubular epithelial cells and renal interstitium, but infrequent or absent in NC group. (Original magnification×400); *P<0.05, IgAN *vs*. NC.

In IgAN group, AIM mainly expressed in glomerular capillary loops and renal tubular epithelial cells, as well as in renal tubular lumen; TGF-β1 expressed mainly in renal tubular epithelial cells, but also in renal interstitium. However, expression of AIM and TGF-β1 were infrequent or absent in normal renal tissues (**Figures 4**, **5**).

### Expression of Macrophages, AIM, TGF-β1 With Clinical Manifestation of IgAN

**Figure 6:**
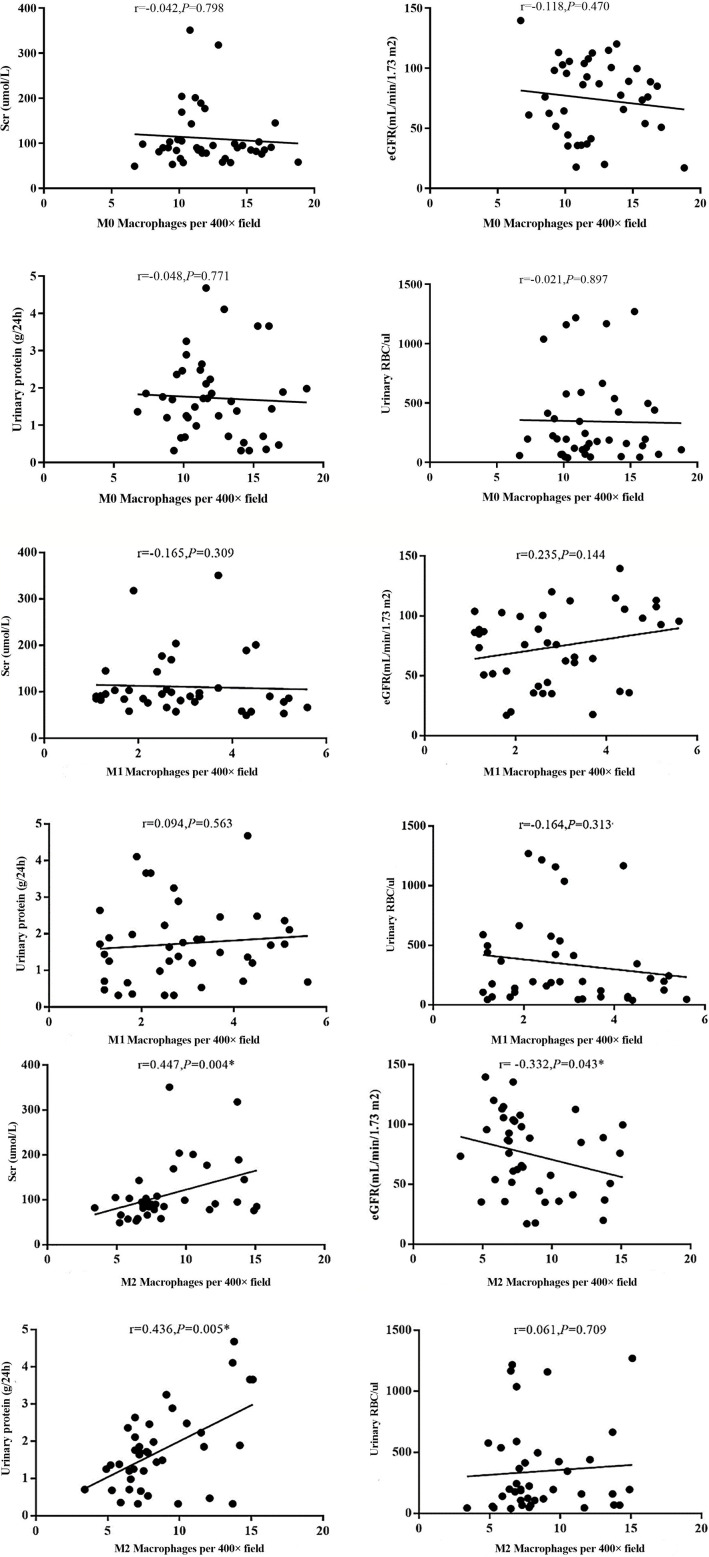
Different types of macrophages and clinical manifestation of IgAN. The number of M2 macrophage infiltration was positively correlated with serum creatinine and 24-hour proteinuria (r=0.447, P=0.004; r=0.436, P=0.005), and negative correlated with eGFR (r=-0.332, P=0.004).

The infiltration of M0 and M1 macrophages in the renal tissue of the IgAN group was not significantly correlated with age, sex, urinary red blood cell count, 24-hour proteinuria and eGFR(*P>*0.05). The number of M2 macrophage infiltration was positively correlated with serum creatinine and 24-hour proteinuria (r=0.447, *P*=0.004; r=0.436, *P*=0.005), and negative correlated with eGFR (r=-0.332, *P*=0.004) (**Figure 6**). 24-hour urinary protein, serum creatinine and eGFR were correlated with AIM and TGF-β1, among which 24-hour urinary protein and serum creatinine were positively correlated with AIM and TGF-β1, while eGFR was negatively correlated with AIM and TGF-β1 (**Table 1**). Since there was no significant correlation between M0 and M1 macrophages and clinical indicators, subsequent data only counted M2 macrophages. Further statistical analysis showed that the expressions of M2 macrophages, AIM and TGF-β1 were different in different pathological types, and there was a correlation among M2 macrophages, AIM and TGF-β1 (**Table 2**).

**Table 1:** Correlation between clinical characteristics and AIM, TGF-β1 of IgAN patients with different pathological stage.

	III (n=17)	IV (n=12)	V (n=11)	*P*	Correlation with AIM	Correlation with TGF-β1
Urinary protein(g/24h)	0.92 ± 0.63	1.50 ± 0.98	3.08 ± 1.62	*P*=0.01	r=0.515, *P<*0.01	r=0.589, *P<*0.01
Scr(μmol/)	79.31 ± 19.73	113.59 ± 24.07	145.40 ± 36.40	*P<*0.01	r=0.748, *P<*0.01	r=0.722, *P<*0.01
eGFR[mL/(min*1.73m2]	94.59 ± 28.05	71.18 ± 19.93	49.84 ± 16.41	*P<*0.01	r=-0.657, *P<*0.01	r=-0.611, *P<*0.01
Urinary RBC(HP)	43.47 ± 45.38	91.49 ± 91.78	88.90 ± 88.59	NS	NS	NS

All data are presented as mean ± SD. NS, no statistical significance.

**Table 2:** AIOD of M2 macrophages, AIM, TGF-β1 among IgAN patients with different pathological stage.

	M2 macrophages	AIM	TGF-β1
	**r** ***P***	**r** ***P***	**r** ***P***
M2 macrophages	— —	0.900 *P*<0.01	0.888 *P*<0.01
AIM	0.900 *P*<0.01	— —	0.913 *P*<0.01
TGF-β1	0.888 *P*<0.01	0.913 *P*<0.01	— —

### Correlation of M2 Macrophages, AIM, TGF-β1, and Fibrotic Area

**Figure 7:**
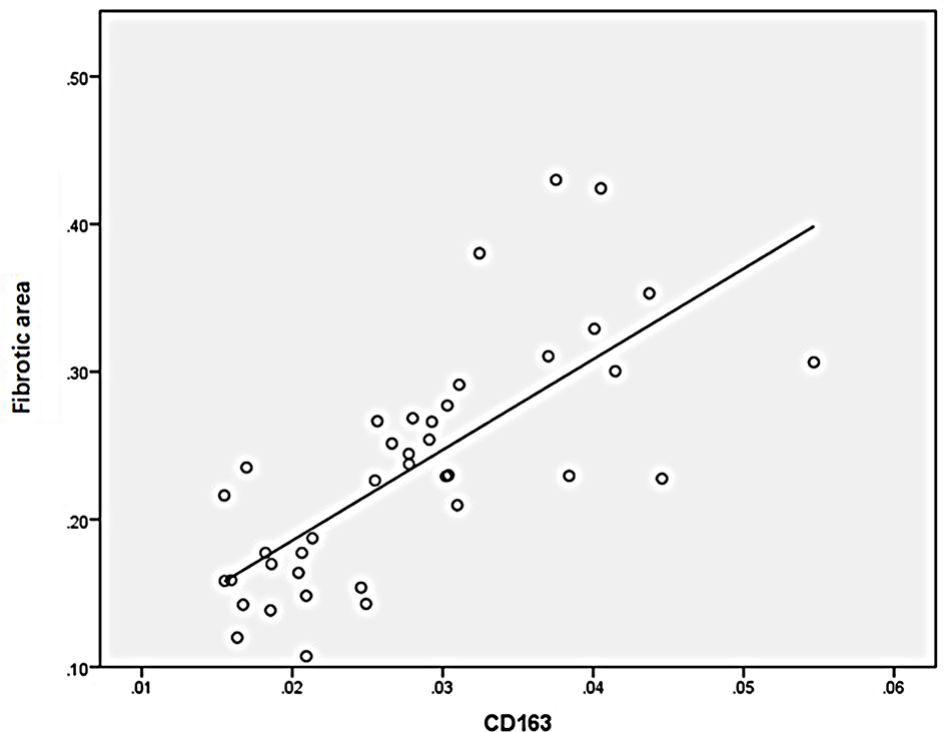
M2(CD163) macrophages were positively correlated with fibrotic area (n=40, r=0.777, P<0.01).

**Figure 8:**
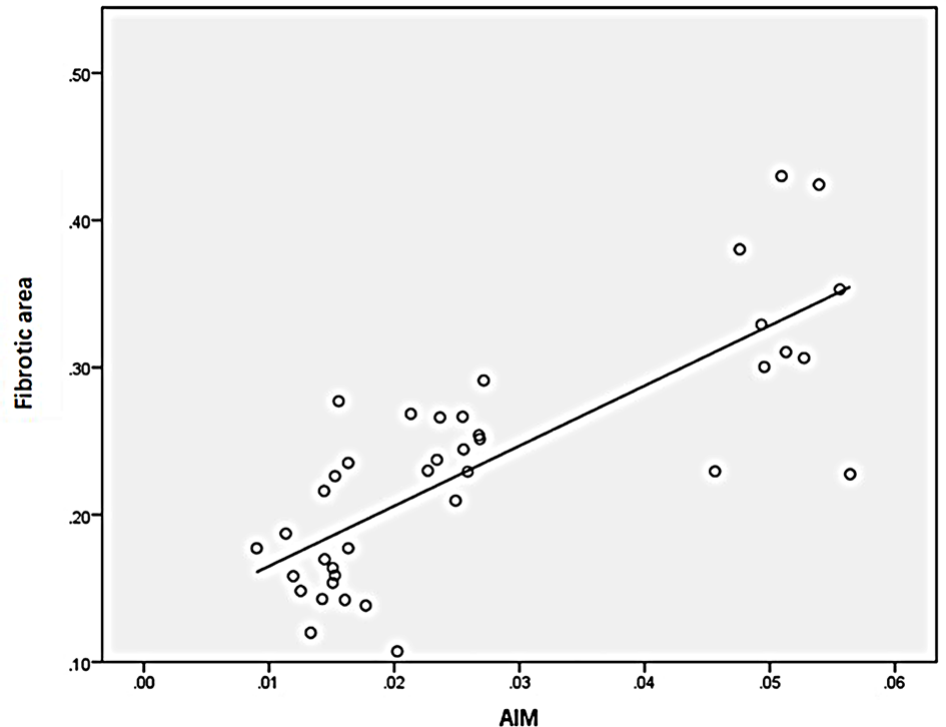
AIM was positively correlated with fibrotic area (n=40, r=0.768, P<0.01).

**Figure 9:**
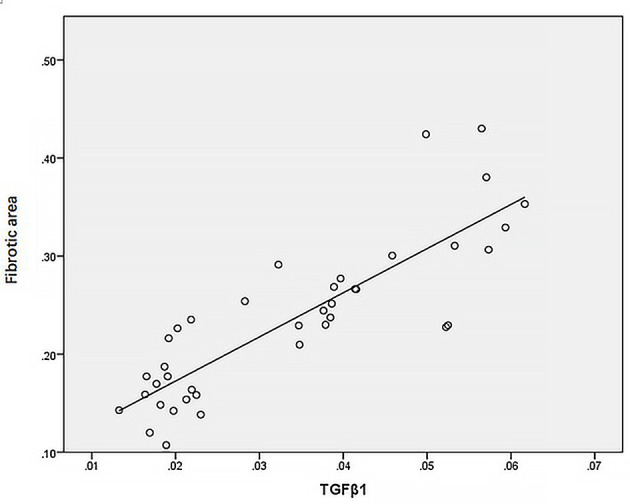
TGF-β1 was positively correlated with fibrotic area (n=40, r=0.853, P<0.01).

Statistical analysis of the correlation between fibrotic area and M2-type macrophages, AIM and TGF-β1 in the tissues of IgAN patients showed that M2 macrophages were positively correlated with fibrotic area (**Figure 7**, r=0.777, *P*<0.01); AIM was positively correlated with fibrotic area (**Figure 8**, r=0.768, *P<*0.01); TGF-β1 was positively correlated with fibrotic area (**Figure 9**, r=0.853, *P<*0.01).

## Discussion

IgAN is the most common primary glomerulonephritis and approximately 40% patients will develop to End-stage renal disease (ESRD) within 20-30 years (21). Renal fibrosis is a necessary process for all CKD develop to ESRD. The pathological manifestations are the formation of a large number of fibroblasts and myofibroblasts and the accumulation of a large number of extracellular matrix, leading to glomerulosclerosis and renal tubular interstitial fibrosis. Ultimately, the renal function loses because of the loss of a large number of normal nephrons (4). At present, renal fibrosis is mainly divided into four stages: 1. The activation of renal tubular epithelial cells and the infiltration of monocytes/macrophages on account of the inflammatory. 2. The release of cytokines, growth factors and other pro-fibrogenic factors. 3. The formation of fibrosis, mainly manifested the deposition of matrix proteins. 4. The deposition of extracellular matrix (ECM), which is a major stage of structural and functional impairment of the kidney (significant reduction of effective nephrons and further decline of glomerular filtration rate).

Myeloid stem cells differentiate into monocytes and enter the circulation from the bone marrow. Monocytes in the circulation migrate and infiltrate into the tissues under different stimuli such as inflammation and trauma, and then differentiate into macrophages. Macrophages are heterogeneous cells in the natural immune system, which can dynamically regulate their phenotypes to adapt to the local microenvironment. Monocytes in the circulation, driven by inflammatory signals, enter the kidney and attach to vascular endothelial cells, induce the release of chemokines and chronic inflammatory factors at the same time. This results in extensive local aggregation of monocytes in renal interstitial, which differentiate into classically activated macrophages (M1) or selectively activated macrophages (M2) by different pathways. At first, pathogens and necrotic cells activate Toll-like receptors and other innate immune receptors, promoting the polarization of M0 macrophages to M1 macrophages. M1 macrophages deposit through the basement membrane at the early stage of kidney injury, promoting a series of inflammatory responses and leading to further kidney injury. As the development of injury, the apoptotic and necrotic cells phagocytosed by macrophages, as well as the subsequent generation of anti-inflammatory factors, promote the polarization of macrophages towards M2 macrophages. M2 macrophages mainly play the roles of anti-inflammatory, promoting renal repair and causing fibrosis (6, 22–26). Therefore, the dynamic balance of the M1 and M2 macrophages indicates the process of inflammation and tissue repair, which determines the prognosis of kidney and drives efforts to therapies targeting macrophages.

In this study, we chose renal pathological tissues of patients who were diagnosed IgAN and observed the polarization of different macrophages. We selected CD68 to represent M0, CD80 to represent M1, and CD163 to represent M2. Our results showed that the number of M0 (CD68^+^) macrophages, M1 (CD80^+^) macrophages, and M2 (CD163^+^) macrophages were respectively significantly increased compared with the normal kidney, indicating that there was a relatively obvious infiltration of macrophages in the renal tissues of IgAN patients.

It was not only found that the infiltration of macrophages in the renal tissue of IgAN patients increased significantly, but also found that there were differences in the number of M0, M1, M2 macrophages in the interstitium of IgAN patients, with more polarization of M0 towards M2. In other words, the infiltration of M2 macrophages is mainly found in the renal tissue of IgAN patients. There was a positive correlation between M2 macrophages and fibrotic area; The distribution of M0 and M1 macrophages in the IgAN group had no significant correlation with age, sex, urinary red blood cell count, 24-hour proteinuria and eGFR (P*>*0.05); However, the number of M2 macrophage infiltration is positively correlated with serum creatinine and 24-hour proteinuria; It is proved that M2 macrophages play a more important role in the progression of IgAN fibrosis.

AIM also plays an important role in many diseases, such as atherosclerosis, non-alcoholic liver disease (16), insulin resistance (27), autoimmune diseases, etc. At the same time, AIM is closely related to renal fibrosis. Megumi Oshima et al. (19) took renal tissue from 43 renal biopsy patients and found that deposition area of AIM and macrophage was positively correlated with the severity of proteinuria and eGFR decrease in patients. It suggested that AIM may have aggravated renal damage. Tadashi Uramat et al. (18)established severe hypertension and induced kidney damage by using a model of spontaneous hypertension tendency (SHRsp) mice. After the treatment of hypertension with related drugs, it was found that the number of macrophages infiltrating into the glomeruli and interstitium was significantly reduced. Compared with the control group, the expressions of AIM and oxLDL were significantly reduced. Moreover, the degree of fibrosis in renal tissue was less than that in the untreated group.

In our study, there were significant differences in the expression of AIM in each pathological grade, and its expression was significantly positively correlated with M2 macrophages, TGF-β1, and the degree of renal fibrosis. This experiment and the above conclusions all suggest that AIM is related to the renal fibrosis process of CKD, which may be related to the anti-apoptotic effect of AIM on macrophages, and then the continuous inflammatory signals promote the further development of fibrosis. Takako Tomita et al. (28) extracted necrotic cells from mice with peritonitis induced by yeast polysaccharides *in vitro*, and found that AIM could promote the phagocytosis of M1 and M2a macrophages to necrotic cells. It was also found in the IgAN model and the acute renal injury model of ischemia reperfusion (IRI) that AIM could promote the clearance of IgA1 and apoptotic cells causing nephritis by macrophages. From the above studies, it is found that AIM, like macrophages, may play different roles in different stages of disease, while the role of AIM in kidney disease may be similar with macrophages.

In a CKD model caused by IRI, M2 macrophages are closely correlated with the process of renal fibrosis, which is caused mainly by secreting TGF-β1 (29). TGF-β1 belongs to the TGF-β superfamily and plays an important role in the development of CKD (30). Tubular epithelial myofibroblast Transdifferentiation (TEMT) is also an important mechanism of renal interstitial fibrosis, and TGF-β1 is an important cytokine regulating TEMT (31). By using TGF-β1 neutralization antibody, inhibitors, gene knockout and other methods to consume TGF-β1, the degree of renal fibrosis can be effectively reduced (32). Up to now, the mechanisms of TGF-β1 causing renal fibrosis mainly include the following points: 1. Induce the synthesis of extracellular matrix, such as collagen I and fibronectin (33). 2. Matrix metalloproteinase (MMPs) and tissue inhibitor of metalloproteinase (TIMPs) are out of balance, thus reducing the degradation of extracellular matrix.3. Promote mesangial cell proliferation, collagen secretion and cause damage to epithelial and podocyte cells, thereby promoting inflammation and further tissue fibrosis (34). 4. Promote the transdifferentiation and proliferation of myofibroblasts from various sources (such as pericytes, epithelial cells, endothelial cells, fibroblasts, macrophages, etc.), thus mediating the development of fibrosis (35). Previous studies have observed that mRNA and protein expression of TGF-β1 in IgAN and the infiltration degree of M2 macrophages in renal tissue were significantly increased compared with normal renal tissue (31), TGF-β1 was co-expressed with M2 macrophages in renal biopsy tissue of CKD induced hypertension. All these suggested that M2 macrophages may play a role in renal fibrosis through TGF-β1.

In this experiment, we found that with the increase of pathological grade, the infiltration of M2 macrophage and the expression degree of AIM and TGF-β1 were also increasing. There was a significant difference among different pathological groups. All the three were closely related to 24-hour urinary protein, serum creatinine, eGFR and other clinical indicators of IgAN patients, and there was a significant positive correlation between the three and the area of renal fibrosis, which indicate that M2 macrophages, AIM and TGF-β1 were all involved in the occurrence and development of IgAN and were closely related to the fibrosis process of IgAN. The results of this experiment were consistent with the study of Braga et al. (36, 37), which found that M2 macrophages are involved in renal fibrosis of unilateral ureteral obstruction (UUO) in a MyD88 signaling pathway. In contrast, studies have found that macrophages play an anti-fibrotic role in the recovery phase of obstructive nephropathy. Nishida et al. (38) demonstrated that angiotensin II type 1 receptor (Agtr1) on interstitial macrophages functions to reduce renal fibrosis at a later stage of UUO. López-Guisa et al. (39) confirmed that macrophages displayed a fibrosis-attenuating role through activating a lysosomal collagen turnover pathway by expressing mannose receptor 2 (Mrc2) in UUO.

M2 macrophages, AIM and TGF-β1 play important roles in the process of IgAN fibrosis, and the three influence each other. However, due to the small samples size of this experiment and the interaction mechanism and relationship among the three, further verification of relevant animal experiments will be needed.

## Data Availability Statement

The raw data supporting the conclusions of this article will be made available by the authors, without undue reservation.

## Ethics Statement

The studies involving human participants were reviewed and approved by Medical Ethics Committee of Subei People’s Hospital of Jiangsu province, Clinical medicine college of Yangzhou University, China. The patients/participants provided their written informed consent to participate in this study.

## Author Contributions

JL and YZ contributed to conception and design of the study, organized the database and performed the statistical analysis. MY wrote the first draft of the manuscript. All authors contributed to the article and approved the submitted version.

## Funding

This research was supported by grants from Jiangsu Commission of Health (Grant No. Z201521).

## Conflict of Interest

The authors declare that the research was conducted in the absence of any commercial or financial relationships that could be construed as a potential conflict of interest.
