# Supplementary figures and images for: The Role of Renal Macrophage, AIM, and TGF-β1 Expression in Renal Fibrosis Progression in IgAN Patients
Source: Front Immunol. 2021 Jun 14;12:646650. doi: 10.3389/fimmu.2021.646650 (PMC8236720; doi:10.3389/fimmu.2021.646650)

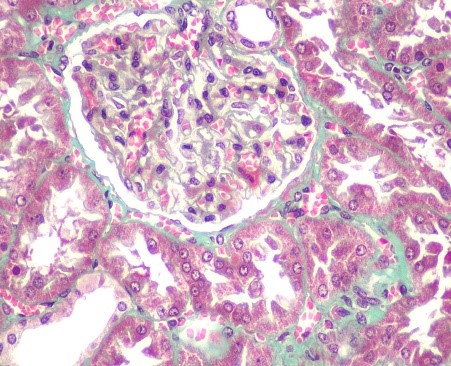

Supplement: Supplementary file 1 [file Image_1.jpeg]

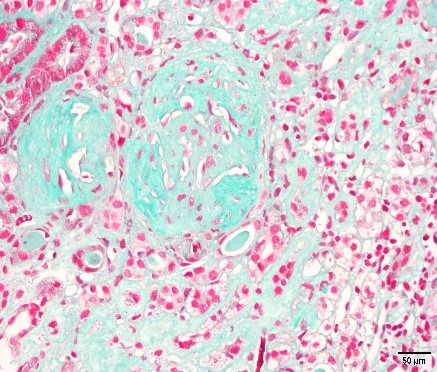

Supplement: Supplementary file 2 [file Image_2.jpeg]
